# Tumor microenvironment-oriented adaptive nanodrugs based on peptide self-assembly

**DOI:** 10.1039/d0sc02937h

**Published:** 2020-07-24

**Authors:** Shukun Li, Wenjia Zhang, Huadan Xue, Ruirui Xing, Xuehai Yan

**Affiliations:** State Key Laboratory of Biochemical Engineering, Institute of Process Engineering, Chinese Academy of Sciences Beijing 100190 China rrxing@ipe.ac.cn yanxh@ipe.ac.cn http://www.yan-assembly.org/; Department of Radiology, Peking Union Medical College Hospital Beijing 100730 China bjdanna95@hotmail.com; School of Chemical Engineering, University of Chinese Academy of Sciences Beijing 100049 China

## Abstract

The aberrant metabolism of tumor cells creates an inimitable microenvironment featuring acidic pH, high glutathione (GSH) levels, and overexpression of certain enzymes, which benefits the overwhelming progress of a tumor. Peptide self-assembly, emerging as a biofriendly and versatile fabrication strategy, harnesses multiple noncovalent interactions to obtain a variety of nanostructures tailored on demand. Orchestrating the reversible nature of noncovalent interactions and abnormal physiological parameters in the tumor microenvironment enables peptide-based nanodrugs to be targetable or switchable, thereby improving the drugs’ bioavailability and optimizing the treatment outcome. This review will focus on peptide-modulated self-assembly of photosensitizers, chemotherapeutic drugs, immunoactive agents for tumor microenvironment-oriented adaptive phototherapy, chemotherapy, immunotherapy and combinatorial therapy. We will emphasize the building block design, the intermolecular interaction principle, adaptive structural transformation in the tumor microenvironment and corresponding therapeutic efficacy, and aim to elucidate the critical role of peptide-modulated, tumor microenvironment-oriented adaptive assemblies in improving the therapeutic index. Challenges and opportunities will be covered as well to advance the development and clinical application of tumor therapies based on peptide self-assembly materials and techniques.

## Abstract 2

The tumor microenvironment is of significance to promote release or reorganization of peptide-modulated nanodrugs, optimizing drug bioavailability and therapeutic outcome.

## Graphical abstract

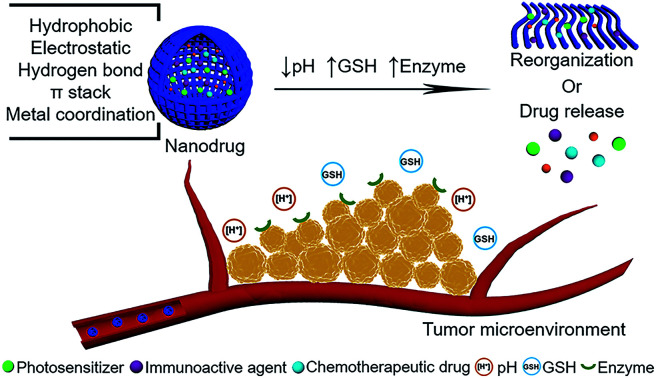

## 1 Introduction

Tumors, as a major burden of disease worldwide, feature a pathophysiological dysfunction of cells and their surrounding environment.^1–3^ The aberrant metabolism of tumor cells creates physiological variations compared to normal tissues and therefore forms a tumor microenvironment, which is inimitably characterized as acidic pH,^4–6^ high glutathione (GSH) concentration,^7–9^ overexpression of certain enzymes or ligands,^10–12^ flawed vascular system and hypoxia,^13,14^ as well as other cells including immune cells, fibroblast cells, and macrophages nourished in an extracellular matrix.^15,16^ The interactions between the tumor and tumor microenvironment are complex and dynamic, which orchestrate cellular or molecular events and ultimately support tumor survival, growth, and metastasis. Therefore, the task of fabrication of smart antitumor drugs with adaptive therapeutic efficacy in the tumor microenvironment is daunting.

Advanced nanotechnology has promoted the development of supramolecular self-assembly for construction of nanodrugs.^17–19^ Peptide self-assembly, in harnessing various noncovalent interactions including hydrophobic interactions, electrostatic forces, hydrogen bonds, π-stacking, van der Waals forces and metal coordination bonds, has emerged as an efficient and powerful strategy for fabrication of smart nanodrugs.^20–22^ Manipulating synergistic or reciprocal noncovalent interactions can obtain tailored nanodrugs. Simultaneously, the reversible nature of noncovalent interactions renders the resulting nanodrugs to be dynamic in terms of structures and functions once they respond to various stimuli (pH,^23–25^ GSH,^26–28^ enzyme,^29–31^*etc.*) in the tumor microenvironment. Such peptide-modulated, tumor microenvironment-oriented adaptive nanodrugs show unique superiorities: (i) with respect to regulation, selecting desired peptide building blocks (hydrophobic, amphiphilic, hydrogen bond-contained, *etc.*) and changing kinetic parameters (temperature, concentration, solvent, *etc.*) yield controlled structures in nanoscale that not only enhance the permeability and retention (EPR) effect,^32^ but also optimize the therapeutic index of drugs. Specifically, the introduction of peptides can modulate the supramolecular photothermal effect of photosensitizers,^33–35^ optimize pharmacokinetic profiles of chemotherapeutic drugs,^36–38^ and form a supramolecular self-adjuvant effect in immunotherapy.^39–41^ (ii) Stimuli-responsive triggers broadly exist in the tumor microenvironment and their significance lie in two aspects. On the one hand, the sensitivity of nanodrugs in the tumor microenvironment is conducive for targeted drug release, permitting optimal therapeutic concentration at tumor sites while eliminating off-target side effects in normal tissues.^42–44^ On the other hand, stimuli in the tumor microenvironment play a switchable role in the morphological reorganization of nanodrugs, which promotes the uptake, the retention, and the efficacy of drugs to a large extent.^45–47^ In particular, integrating targetability and switchability may be a promising alternative for further improving treatment outcome. (iii) Peptides are naturally-occurring building blocks with controllability, programmability, and biosafety.^48–50^ Owing to the biological capability of peptides fulfilled in organisms, introducing the biofunction of peptides into nanodrugs is easily achieved.^51–53^ For example, the immune bioactive peptides encompassing specific amino acid sequences that act as antigens, adjuvants and checkpoint blockades, have been recently studied as building blocks to construct nanodrugs for combinational antitumor therapy.^54–56^ This paradigm avoids the addition of ineffective components, simplifying the design and amplifying the therapeutic effect. However, elaboration of the relationship between adaptive assembly and function implementation, intrinsically the dynamic control of noncovalent interactions in the tumor microenvironment, still remains a challenge. Therefore, a perspective on peptide-modulated self-assembled nanodrugs with adaptive therapeutic functions in the tumor microenvironment is imperative to advance the further development of nanodrugs.

**Fig. 1:**
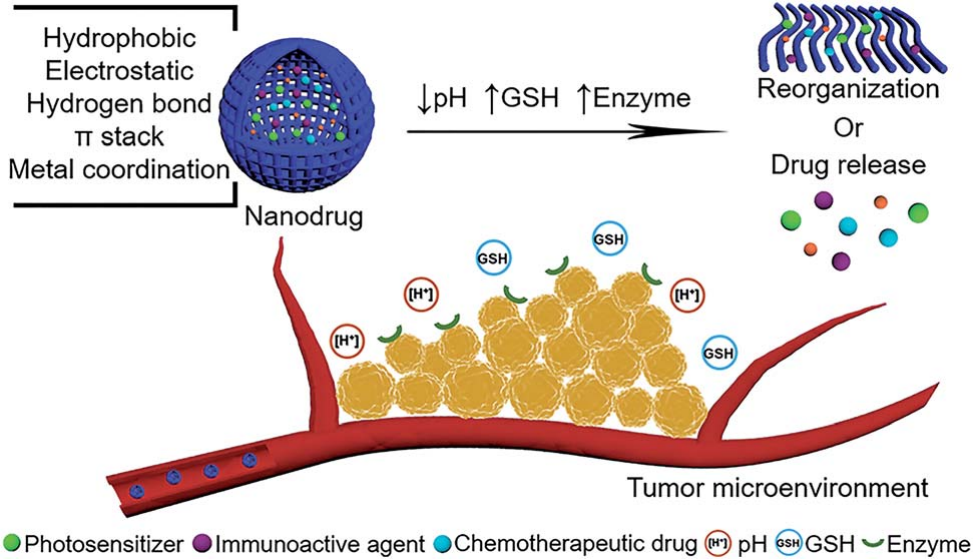
Schematic illustration of peptide-modulated, tumor microenvironment (pH, GSH, enzyme)-oriented adaptive nanodrugs for tumor therapy.

In this review, we will focus on the peptide-modulated self-assembly of photosensitizers, chemotherapeutic drugs and immunoactive agents for tumor microenvironment-oriented adaptive (1) phototherapy, (2) chemotherapy, (3) immunotherapy and even (4) combinatorial therapy (Fig. 1). The emphasis will put on building block design and intermolecular interaction principle in the tumor microenvironment and the corresponding function implementation, aiming to elucidate the critical roles of peptide-regulated, tumor microenvironment-oriented adaptive assemblies in improving the therapeutic index. Representative examples with tumor microenvironment (pH, GSH, enzyme)-responsiveness based on the above therapies will be presented, providing unique perspectives to advance the development of nanodrugs. Finally, we will lay out challenges and opportunities for the utilization of peptide-modulated, tumor microenvironment-oriented nanodrugs to optimize preclinical tests and accelerate their final clinical applications.

## 2 Phototherapy

Phototherapy, mainly including photodynamic therapy (PDT) and photothermal therapy (PTT), relies on the excitation of photosensitive molecules through light to generate reactive oxygen species (ROS) or local hyperthermia to kill tumor cells. Intriguingly, photosensitive molecules not only act as therapeutic drugs but also integrate diagnosis function, permitting them to be theranostic agents. Peptide self-assembly has become a facile method for fabrication of photosensitive theranostic nanodrugs. In essence, modulating the photophysical properties of photosensitive molecules by peptide self-assembly determines the therapeutic and imaging modalities. In the Jablonski diagram, the light excited photosensitive molecules decay back to the ground state through three pathways: fluorescence emission (fluorescence imaging), intersystem crossing to the triplet excited state followed by energy transfer with adjacent oxygen to generate reactive oxygen (ROS) species (PDT), and thermal deactivation *via* nonradiative relaxation (PTT and photoacoustic imaging). Normally, the excited photosensitive molecules in the molecular state are prone to emit fluorescence with accompanying intersystem crossing that generates ROS. Once the photosensitive molecules were modulated into an aggregation state, the absorbed energy competitively dissipated through thermal relaxation. Therefore, the peptide-modulated photosensitive self-assemblies possess a supramolecular photothermal effect that can be applied for photoacoustic imaging and PTT. Combined with tumor microenvironment-oriented drug release or morphological reorganization, the resulting nanodrugs may restore their capabilities of fluorescence emission and photodynamic effect, thereby maximizing therapeutic effect.

### 2.1 pH

The disordered metabolism of tumor cells contributes to the acidic microenvironment.^57^ Compared to the physiological pH 7.4 in blood and normal tissues, more acidic pH values are found in interstitial tumors (pH 6.5–7.2) and endosomes (pH 5.0–6.5), which can be exploited as triggers for photosensitive drug release to ensure the optimal concentration at tumor sites, especially favoring for fluorescence imaging and PDT.

Protonation of building blocks occurring in the acidic tumor microenvironment is beneficial for targeted drug release. For example, our group^58^ co-assembled a short peptide (H-Phe-Phe-NH_2_·HCl, CDP) or an amino acid derivative (9-fluorenylmethoxycarbonyl-l-lysine, Fmoc-l-Lys) with chlorin e6 (Ce6) to form photosensitive nanodrugs. The aromatic groups in CDP and the Fmoc groups in Fmoc-l-Lys can interact with the pyrrole rings in Ce6 through hydrophobic interactions or π–π stacking, which promoted the formation of Fmoc-l-Lys/Ce6 nanodrugs and CDP/Ce6 nanodrugs. Compared to free Ce6, higher tumor-selectivity with Fmoc-l-Lys/Ce6 nanodrugs and CDP/Ce6 nanodrugs was obtained due to the EPR effect. Once they reached into the tumor microenvironment, protonation of the carboxyl groups in Ce6 molecules occurred, thus weakening the electrostatic interactions of the building blocks, leading to disassembly of Ce6 molecules, achieving imaging-guided PDT. In another example, Yan and coworkers^59^ conjugated a chemotherapeutic drug (mannose) to a pH-sensitive polypeptide copolymer (PE) through formation of Schiff base bonds, obtaining a pH-sensitive mannose-containing copolymer (PM). The photosensitive molecule iodinated boron dipyrromethene (BDPI) was encapsulated to obtain the nanodrug. In the acidic tumor microenvironment, the detachment of the Schiff base bonds and the protonation of the tertiary amine group in PM destroyed the nanostructure, resulting in release of BDPI and mannose for PDT and chemotherapy. Alternatively, acidic pH can cleave some specific bonds to achieve drug release. For instance, hydrazone and benzoic-imine are typical pH-responsive bonds, which are widely utilized in the design of targeted drug release in the tumor microenvironment.^45^

Besides targeted drug release, the structural transformation of nanodrugs can also be oriented by the tumor acidic microenvironment. Yu and coworkers^60^ reported that pH-responsive isomerization spatially optimized cellular uptake of nanodrugs. In the design, Ce6 was conjugated to a pentapeptide to obtain AmpF-C. The 4-amino-proline (Amp) amide bonds contained in AmpF-C showed pH-responsive isomerization. In solution at pH 7.4, AmpF-C self-assembled into superhelices with a height of approximately 7 nm. Decreasing the solution pH to 6.5 and 5.5 resulted in the *cis* to *trans* isomerization of the Amp amide bonds, promoting the transformation of AmpF-C from superhelices to nanoparticles with diameters of 104.1 ± 14.6 nm, and 120.2 ± 17.2 nm, respectively. Such a pH gradient is parallel to that in the tumor microenvironment, implying their potential for endosome/lysosome-involved cellular uptake and PDT. Moreover, other pH-responsive structural transformations are able to optimize delivery processes. For instance, the size decrease that nanodrugs undergo in response to acidic pH in the tumor microenvironment can overcome the diffusion hindrance.^47^ The pH-responsive shape switch from spherical nanodrugs to rod-like nanodrugs results in enhancement of the cellular internalization.^61^

Surface charge is of significance in determining the fate of nanodrugs *in vivo*. An ideal nanodrug should be negatively or neutrally charged during blood circulation as to avoid clearance by the reticuloendothelial system, while it should be positively charged in tumor tissues to enhance the cellular uptake. The “charge dilemma” was eliminated by the strategy of tumor acidity-specific charge reversal nanodrugs developed by Wang and coworkers.^62^ Generally, tumor acidity-cleavable maleic acid amide (TACMAA) was incorporated in the design, which was produced by a reaction of an amino group with 2,3-dimethylmaleic anhydride (DMMA). In the acidic tumor microenvironment, nanodrugs containing TACMAA realized their charge switching for cellular uptake enhancement. An expanded molecule of 2-propionic-3-methylmaleic anhydride (CDM) was utilised in the fabrication of the pH-facilitated charge reversal nanodrug, D_m_-NP.^63^ The building block of PEG-Dlink_m_-R9-PCL encompassed a poly(ethylene glycol) (PEG) corona for prolonging circulation time, a pH-responsive amide bond as a Dlink_m_ linker, and a cell-penetration peptide poly(ε-caprolactone)-R9 (PCL-R9), and self-assembled into micelles. Importantly, the R9 in the hydrophilic shell of D_m_-NP allowed for the encapsulation of siRNA to form micelleplexes, D_m_-NP_siN.C._, with a surface charge of 23.9 mV at pH 7.4. Decreasing the pH to 6.8, the Dlink_m_ linker was cleaved to remove the PEG corona and the PCL-R9 was exposed to transform the surface charge of D_m_-NP_siN.C._ into 34.8 mV, which facilitated the targeted cellular uptake in tumor tissues, and achieved superior tumor inhibition activity. Adopting the same tumor acidity induced charge reversal strategy, Yang and coworkers^64^ used DMMA to modify amines in TAT lysine residues to avoid clearance during blood circulation. The photosensitizer Ce6 and contrast agent Gd^3+^ were encapsulated into the modified TAT and formed ^DA^TAT-NP nanostructures. Once accumulated in the acidic tumor microenvironment, the DMMA groups were detached from ^DA^TAT-NP. The exposed TAT enhanced cell penetration and cellular uptake, resulting in enlarged fluorescence and MR signals for imaging-guided PDT.

### 2.2 GSH

Redox potential is a characteristic stimulus in the tumor microenvironment. The concentration of GSH at tumor sites is fourfold higher than that in normal physiological conditions. Furthermore, the concentration of GSH inside tumor cells is almost three orders of magnitude higher than that in the extracellular plasma. Therefore, the differences between GSH concentrations pave avenues for the design of peptide-modulated, GSH-oriented adaptive nanodrugs.

Taking advantage of the evaluated GSH concentration, disulfide bonds become the most common chemical bonds that are incorporated into nanodrugs. Our group^65^ have developed disulfide bonds cross-linked cationic dipeptide (CDP)-based nanodrugs. Electrostatic interactions and hydrophobic interactions promoted the co-assembly of CDP and Ce6. Glutaraldehyde assisted the co-assemblies to form disulfide bonds to improve the structural stability. Importantly, GSH in the tumor microenvironment broke the disulfide bonds in nanodrugs, achieving Ce6 release for PDT. Similarly, as another example of disulfide bonds that respond to GSH in the tumor microenvironment, Tan and coworkers^66^ designed a pH (low) insertion peptide (pHLIP) for a pH_e_-driven targeting conjugate, Ce6-pHLIP_ss_-AuNRs. pHLIP was a class of peptide that can fold and insert cross membrane at an acidic pH value,^67^ leading to a unique way for targeted drug delivery. In the design, a side peptide chain containing cysteine residues was conjugated to the pHLIP by a disulfide bond to obtain pHLIP_ss_. Then, the Ce6-pHLIP_ss_ was synthesized. Finally, the Ce6-pHLIP_ss_ and thiol-terminated monomethoxyl poly(ethylene glycol) (mPEG-SH) were assembled on the surface of AuNRs by thiol chemistry, obtaining Ce6-pHLIP_ss_-AuNRs. AuNRs acted as a photothermal agent and Ce6 acted as a photodynamic agent. The reduction of the disulfide bond in the conjugate by extracellular GSH in the tumor microenvironment enabled separation of Ce6 from Ce6-pHLIP_ss_-AuNRs. Owing to the pH_e_-driven targeting ability and GSH-driven activated PDT efficacy, the accumulation of Ce6-pHLIP_ss_-AuNRs was enhanced and a better synergistic efficacy of PDT and PTT was achieved.

**Fig. 2:**
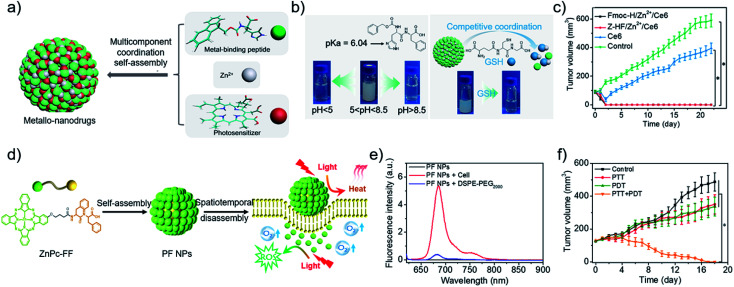
(a) Construction of metallo-nanodrugs through multicomponent (small peptides, photosensitizers, zinc ions) coordination self-assembly. (b) Ultrasensitive responsiveness of metallo-nanodrugs to pH and GSH. (c) Tumor growth profiles of mice after PDT. Reproduced with permission from ref. 7. Copyright 2018 American Chemical Society. (d) Spatiotemporally coupled photoactivity of PF self-assemblies for localized adaptive tumor theranostics. (e) Fluorescence spectra of PF NPs in the presence of DSPE-PEG_2000_ and in cell suspension. (f) Tumor growth profiles of mice after phototherapy. Reproduced with permission from ref. 42. Copyright 2019 Wiley VCH.

Metal ions coordination in living organisms is critical for aspects of structure and function, which guides the fabrication of delicate nanostructures.^68^ More recently, our group^7^ developed metallo-nanodrugs based on multicomponent metal coordination self-assembly (Fig. 2a), which is inspired by hemoglobin that integrates porphyrin derivatives and histidine residues *via* cooperative coordination. A histidine-containing dipeptide (*N*-benzyloxycarbonyl-l-histidine-l-phenylalanine, Z-HF) and amphiphilic histidine derivative (9-fluorenylmethoxycarbonyl-l-histidine, Fmoc-H) were selected as building blocks. Carboxyl group-containing Ce6 was selected as a model photosensitive drug. Zinc ions coordination self-assembly with imidazole rings in the peptide and carboxyl groups in Ce6, together with hydrophobic interactions and π–π stacking, promoted the formation of metallo-nanodrugs, Fmoc-H/Zn^2+^/Ce6 (79 ± 21 nm) and Z-HF/Zn^2+^/Ce6 (76 ± 21 nm). Acidic pH rendered the protonation of imidazole rings and high GSH competitively coordinated with zinc ions within the metallo-nanodrugs (Fig. 2b). Dual-stimuli jointly weakened the metal coordination interactions of the nanodrugs, which promoted their disassembly in the tumor microenvironment. The disassembled Ce6 molecules were conducive for fluorescence imaging and PDT effects. Since structural stability in blood circulation and ultra-sensitive responsiveness in the tumor microenvironment were integrated into the metallo-nanodrugs, tumors in mice injected with the metallo-nanodrugs were eradicated (Fig. 2c). As another optimization strategy, our group^26,69^ introduced the imaging metal ion Mn^2+^ that not only regulated structural stability but also bestowed the nanodrugs with magnetic resonance imaging (MRI) properties. Notably, exploitation of GSH cooperative coordination in the tumor microenvironment realized the dynamic disassembly of the nanodrugs. Meanwhile, it eliminated the intracellular GSH that is detrimental to ROS generation.

### 2.3 Enzyme

Tumor cells overexpress certain enzymes, such as matrix metalloproteinases (MMP), alkaline phosphatases (ALP) and carboxylesterases (CES), which can cleave their corresponding substrates in peptide-based building blocks, leading to enzyme-induced drug release or morphological transformation of self-assemblies.

Enzyme-instructed peptide self-assembly was pioneered by Xu and coworkers.^70,71^ A variety of hydrogelator-precursors with distinct enzyme responsiveness were designed and synthesized. Rao and coworkers^72^ extended the concept as they employed a bioorthogonal cyclization reaction to control the self-assembly of small molecules and suggested its application in tumor enzyme activity. Wang and coworkers^73^ applied enzyme-assisted peptide self-assembly in phototherapy. For example, they designed and synthesized a peptide–photosensitizer conjugate (compound **1**) that was composed of three parts: photosensitizer (Purpurin 18, P18), gelatinase-responsive linker PLGVRG, and a targeting ligand RGD that bind to α_v_β_3_ integrin on tumor cell membranes. Compound **1** diffused and extravasated readily in blood circulation. RGD promoted the circulation of compound **1** to the tumor microenvironment, and then the gelatinase overexpressed by tumor cells cleaved the PLGVRG linker. The enhanced hydrophobic interactions and the reduced steric hindrance resulted in the self-assembly of building blocks for the formation of nanofibers. Such nanofibers exhibited prolonged retention time and led to an enhanced photoacoustic signal and therapeutic efficacy. Zhang and coworkers^74^ synthesized another peptide–photosensitizer conjugate for tumor-targeting PDT. A cell-penetrating peptide (R_9_GPLGLAGE_8_, ACPP) is sensitive to MMP-2 that is overexpressed in the tumor microenvironment. Protoporphyrin (PpIX), as the photosensitizer, was conjugated to ACPP. In normal tissue, the polyanionic peptide (E_8_) will block the cell-penetrating function of polycationic CPP (R_9_) through intramolecular electrostatic interactions. Once they circulated to the tumor site, proteolysis of the oligopeptide linker (GPLGLAG) between R_9_ and E_8_ occurred, disassociating R_9_-PpIX from E_8_. ACPP-PpIX remained stable in blood circulation, while MMP-2 overexpressed on tumor cells can orient the targeted release of R_9_-PpIX for effective PDT.

Effective PDT requires the photosensitizer to be kept in a molecular state. By contrast, a considerable aggregation state of photothermal nanodrugs is preferred as to achieve thermal relaxation for PTT. A supramolecular effect can be readily realized by aggregating photosensitizer at the tumor site.^33,34,75^ Joining the self-assembly and tumor microenvironment-oriented disassembly of photosensitizers readily achieves the combination of PTT and PDT. For instance, our group^42^ designed and synthesized a phthalocyanine–peptide conjugate (PF). Driven by the hydrophobic interactions and π–π stacking, PF self-assembled into nanoparticles (PF NPs) with a diameter of 54.8 ± 17.6 nm. Simultaneously, supramolecular photothermal effect was generated for PTT. While PF NPs interacted with the hydrophobic domains widely existing on the cell membrane, PF NPs disassembled into molecules for PDT (Fig. 2d), as evidenced by the restored fluorescence intensity of molecular PF conjugates (Fig. 2e). The adaptive transformation of the phthalocyanine–peptide conjugate in the tumor microenvironment enabled the integration of multiple therapeutic modalities in one component, achieving a superior localized antitumor effect (Fig. 2f). The strategy is facile and effective, however, improvement in the targeting ability of the PF conjugate should be the next research interest.

## 3 Chemotherapy

Chemotherapy is a traditional therapeutic modality in clinical use. Unfortunately, the treatment efficacy of chemotherapy is severely impeded by the occurrence of acquired multidrug resistance (MDR) in tumor cells and side effects to normal tissues, which are caused by insufficient drug concentration in tumor sites and premature drug release during whole body blood circulation. Peptide-modulated, tumor microenvironment-oriented chemotherapeutic self-assemblies possessing structural controllability, high loading efficiency, extended blood circulation and targeting ability tackled the above problems and thus optimized therapeutic outcome.

### 3.1 pH

Chilkoti and coworkers^76^ reported a pH-dependent nanodrug that assembled by a chimeric polypeptide–doxorubicin (DOX) conjugate to abolish tumor cells *via* a single injection. A pH-responsive hydrazone bond linked the polypeptide and DOX. The hydrophobic DOX and hydrophilic polypeptide imparted sufficient amphiphilicity to the conjugates that promoted their self-assembly into a nanodrug. Intriguingly, the hydrazone bond was considerably stable during blood circulation at pH 7.4. While with decreased pH at 5.0 which is relevant to endosome/lysosomal trafficking in the tumor microenvironment, the hydrazone bond was cleaved and DOX was liberated for effective chemotherapy. Additionally, a pH-sensitive switchable nanodrug was reported by Hu and coworkers.^77^ DOX molecules were encapsulated into liposomes (LS), and the LS were functionalized by a peptide STP (SKDEEWHKNNFPLSP). In the presence of protons within the acidic tumor microenvironment, the segment of KDEE in STP can form an α helix, which could improve the internalization. STP also acted as an affinity ligand for vessel marker endothelial growth factor receptor-2 (VEGFR-2) on the tumor, hence the formed STP-LS-DOX nanodrugs possessed the dual function of pH-triggered responsiveness and VEGFR-2-oriented targetability. Therefore, the STP-LS-DOX nanodrugs could enhance the antitumor effect.

Normally, the therapeutic efficacy of chemotherapeutic drug is determined by five steps: including circulation, accumulation, retention, internalization, and release. In this regard, Chen and coworkers^37^ developed a hierarchical responsive strategy to enhance the above five steps of nanodrugs. A peptide conjugate, RGD-PC7A-POEG-PssCPT, was designed. Cyclic RGD peptide was applied as the targeting segment to bind to the tumor membrane. The triblock copolymer included a hydrophobic PC7A chain, hydrophilic poly(oligo-(ethylene glycol) monomethyl ether methacrylate) (POEG) segment and hydrophobic poly reduction-responsive camptothecin (CPT) prodrug (PssCPT) chain. During blood circulation at the neutral pH of 7.4, the RGD-PC7A and PssCPT segments were trapped inside the hydrophobic core and POEG was coated at the surface of the nanodrug, thus the targeting ability of RGD was shielded and the circulation time of the nanodrug was prolonged. While in the tumor microenvironment with decreased pH below 6.8, protonation of PC7A occurred and POEG became positively charged, resulting in exposure of RGD in the nanodrug. RGD oriented the nanodrug to bind on the cell membrane and therefore enhanced its tumor retention and cellular internalization. Furthermore, the high concentration of GSH within cells cleaved the disulfide bond in PssCPT, further promoting release of CPT molecules. Such features-packed nanodrugs have demonstrated their targetability, potency and safety *in vivo*.

Exploiting the toxicity of peptide drugs may be another method to improve the therapeutic index. Cytotoxic peptide (KLAKLAK)_2_ (KLAK) is a proapoptotic peptide that can disrupt mitochondria structures and then induce apoptosis. Wang and coworkers^78^ synthesized amphiphilic hyperbranched poly(β-thioester)s (PPHD-PK) conjugated with KLAK and PEG. In aqueous solution, PPHD-PK self-assembled into nanoparticles, whereas PPHD acted as an inner core for encapsulation of DOX, PEG and KLAK were displayed as the hydrophilic outer shell. Acidity in the tumor microenvironment broke the β-thiopropionate group in PPHD to induce release of DOX and KLAK, leading to potent efficacy for killing tumor cells. As a more precise strategy *in vivo*, they^79^ recently designed a peptide conjugate with the property of acidity-increased hydrophobicity in the tumor microenvironment. KLAK was decorated with an acid-responsive moiety *cis*-aconitic anhydride (CAA). Cell-penetrating peptide TAT was conjugated to the poly(β-thioester)s backbones. Thus, PT-K-CAA was obtained that was negatively charged and kept as a single chain at pH 7.4, which can penetrate deeply into a tumor. Intriguingly, the hydrolysis of CAA in the tumor microenvironment at pH 6.5 led to increased hydrophobicity of the peptide conjugate, which enforced their self-assembly into nanoparticles. The strategy enhanced internalization efficiency and promoted therapeutic efficacy of KLAK for killing deep solid tumors.

### 3.2 GSH

Metal coordination-driven multicomponent self-assembly, as a novel fabrication of nanodrugs, is also applicable in chemotherapy to improve circulation stability and achieve targeted release of chemotherapeutic drugs. Our group^36^ demonstrated that curcumin-based nanodrugs can be assembled through the multicomponent coordination interactions of curcumin, zinc ions and metal-binding peptides or amino acids. Intriguingly, the size of nanodrugs can be controlled through regulation of kinetic parameters (tension coefficients of the solvent) or dynamic parameters (concentration of curcumin) in the process of preparation. Concurrently, a high loading efficiency is readily obtained (∼52%). Moreover, competitive ligands of GSH can re-coordinate with zinc ions, resulting in curcumin release for cellular uptake enhancement and chemotherapy improvement.

**Fig. 3:**
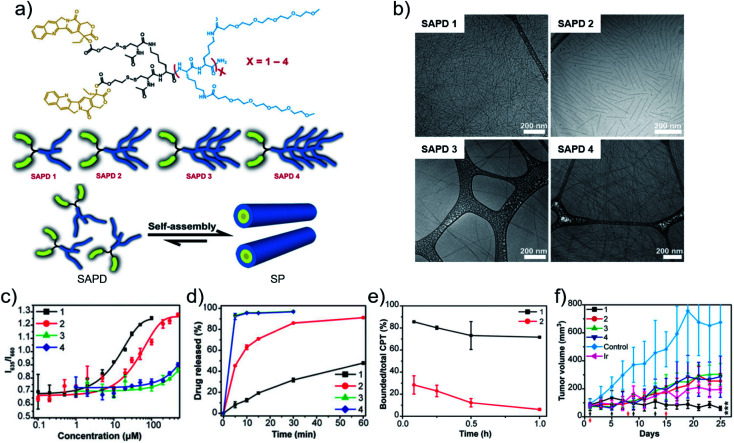
(a) Chemical structures, cartoons of the designed self-assembling prodrugs (**SAPD**s) and schematic illustration of self-assembly of **SAPD** into supramolecular polymer (SP). (b) Cryo-TEM images of supramolecular assemblies of **SAPD**s. (c) CMC measurement of **SAPD**s using a Nile red method. (d) Drug release profiles of **SAPD**s in rat plasma with 10 mM GSH. (e) Profiles of the ratio of bound CPT in **SAPD**s to total CPT after injection. (f) Tumor volume profiles of mice after chemotherapy. Reproduced with permission from ref. 81. Copyright 2020 National Academy of Sciences.

As another flexible method to modulate chemotherapeutic drugs self-assembly, amphiphilic peptide–drug conjugates, named as prodrugs, were developed by Cui and coworkers.^80^ The design endowed chemotherapeutic drugs with the dual capabilities of regulation and chemoactivity. More recently, they suggested the significant role of critical micellization concentration (CMC) in designing and developing chemotherapeutic drug delivery systems.^81^ Four prodrugs (**SAPD**s) were synthesized, which consisted of two hydrophobic CPT molecules and oligoethylene-glycol (OEG)-decorated peptides with various OEG numbers of 2, 4, 6, 8, named as **SAPD** 1, **SAPD** 2, **SAPD** 3, and **SAPD** 4, respectively. In addition, the CPT and the hydrophilic moiety were connected *via* a GSH-responsive disulfanyl-ethyl carbonate linker (etcSS) (Fig. 3a). Driven by the directional π–π stacking, self-assembled nanodrugs were obtained. **SAPD** 1 formed supramolecular filaments with a diameter of 9 nm which were several micrometers in length. **SAPD** 2 formed shorter filaments with a length less than 400 nm. **SAPD**s 3 and 4 both aggregated into micrometer-long nanoribbons of various widths because of the increase in hydrogen bonds supplied by OEG (Fig. 3b). The CMC values were estimated to be 2.7 and 10.1 μM for **SAPD**s 1 and 2, respectively, while the CMCs of **SAPD**s 3 and 4 exceeded 200 μM and cannot be accurately extracted (Fig. 3c). The results implied the structural stability of the **SAPD** SPs would be **SAPD** 1 > **SAPD** 2 > **SAPD**s 3 and 4. In the presence of GSH (10 mM), the drug release rate was **SAPD** 1 < **SAPD** 2 < **SAPD**s 3 and 4 (Fig. 3d), which may be attributed to the fact that the more stable the structure, the less the drug release. An *in vivo* circulation study revealed that **SAPD** 1 maintained a lower degradation in plasma (86% remained in the bound form) compared to **SAPD** 2 (28% remained in the bound form) within 5 min (Fig. 3e), implying the importance of the CMC in determining the morphological and structural integrity of supramolecular self-assemblies in blood circulation. With the enhanced stability and prolonged circulation time, the antitumor effect of **SAPD** 1 was superior to other groups (Fig. 3f). The work established a correlation between the CMC of drug molecules and their *in vivo* performance. Similarly, Cao and coworkers^82^ also investigated the important role of the CMC of drug molecules in supramolecular systems for improving targeting and therapeutic outcome.

### 3.3 Enzyme

Enzyme-instructed drug–peptide amphiphilic hydrogelators can be developed as hydrogel networks for enhancing targetability and uptake of nanodrugs in the tumor microenvironment. Motivated by the principle, Xu and coworkers^83^ have developed a tumor intracellular self-assembly strategy harnessing CES to overcome drug resistance. An enantiomeric pair of precursors containing taurine, l-DPT and d-DPT, were synthesized, where the naphthyl capped diphenylalanine at the N-terminal provided self-assembly forces including π–π stacking and hydrogen bonds. After the hydrolysis by CES overexpressed in tumor cells, the taurine moiety was detached and the hydrogelators, l-DP and d-DP, were obtained. Then, the hydrogelators self-assembled to form nanofibrils, which disrupted the dynamics of actin filaments in tumor cells and thus caused their necroptosis or apoptosis. Particularly, compared to normal cells, the inhibition capacity for tumor cells was specific. Additionally, the inhibition of tumor cells was selective, which was positively correlated with the CES activity in various tumor cells. The expression of enzymes, such as CES and ALP, differs in different cell lines. For example, comparing the two cell lines OVSAHO and HepG2, the activities of ALP overexpressed by them are comparable, while the activity of CES in the HepG2 cell line is higher than that in the OVSAHO cell line. The difference prompted Xu and coworkers^84^ to couple the assembly–disassembly dynamic process for selectively killing tumor cells that downregulated CES. They designed peptide-based precursors as the substrates of both CES and ALP. The peptide-based precursors can turn into building blocks after ALP hydrolysis and self-assemble into nanofibrils. In tumor cells that upregulated CES, hydrolysis of ester bonds in the building blocks enabled disassembly of the nanofibrils, thus being innocuous to the tumor. By contrast, in tumor cells with downregulation or dysfunction of CES, there was no occurrence of hydrolysis of the nanofibrils, hence the nanofibers caused tumor cell death.

Gianneschi and coworkers^85^ exploited the MMP overexpressed in an array of tumor types as a targeting tool for the delivery of chemotherapeutic drugs. They designed a peptide–paclitaxel conjugate, where the peptide is hydrophilic and can respond to MMP. The amphiphilic conjugate can self-assemble into micellar nanodrugs with a peptide shell and drug core due to the hydrophobic interaction. Notably, the peptide shell was cleaved upon exposure to MMP in the tumor microenvironment, then the nanodrugs underwent a drastic change in morphology from discrete, spherical micelles with a diameter of 20 nm to form micrometer scale assemblies, leading to release of PTX and achieving a measurable therapeutic dose for effective chemotherapy. Despite the tremendous successes of MMP-responsive nanodrugs for chemotherapy, however, the responsiveness is still hindered by the accessibility of the MMP to the substrates that are often embedded inside the assembled structures. Although the resistance of the assembled structures to enzymatic degradation was improved, inevitably, the responsiveness of the assembled nanostructures to MMP is less sensitive than their corresponding monomeric molecules. To address this problem, Cui and coworkers^86^ developed a strategy of post-assembly crosslinking to promote the formation of enzyme–substrate complexes and further to facilitate the enzymatic reaction. The synthesized amphiphilic peptide MASP1 self-assembled into filaments at pH 4.5, then, MMP-2 specific peptide substrates were utilized to crosslink above the filaments. Of note, in the presence of MMP and at pH 7.5, peptide substrates on the filament surface were specifically cleaved by MMP, disassociating the cross-linked filaments. Integrating the improved stability and enzyme-induced instability at targeted sites, the strategy implied its great potential as drug carriers. From the perspective of molecular design, they^87^ demonstrated the significance of the unsymmetric reverse bolaamphiphiles (RBA) design in exposing the MMP-2 cleavable segments on the filament surface. The reverse peptide bolaamphiphile consists of four parts. A hydrophilic peptide sequence RGDR and an MMP cleavable peptide sequence PLGVR were in the middle, while two structurally different hydrophobic segments, a triple valine sequence and a twelve-carbon alkyl chain, were capped at the C-terminus and N-terminus, respectively. Hydrogen bonds and hydrophobic interactions promoted the RBA to self-assemble into supramolecular filaments with MMP-2 cleavable sequence displayed on the surface, and then to entangle into a hydrogel. For the utilization of the design in chemotherapy, paclitaxel was conjugated to the N-terminus instead of the alkyl tail. Furthermore, a reducible linker buSS was used to link the paclitaxel and peptide. In a tumor microenvironment that overexpressed MMP-2 and enriched GSH, the supramolecular hydrogel was degraded into fragments and then into monomers, leading to the drug release for effective chemotherapy.

Enzyme-triggered morphology transitions are implicated in biological functions. For instance, MMP-2 triggered fiber-to-micelle morphological transition of self-assemblies enhanced their cell-penetrating ability as reported by Azevedo and coworkers.^88^ Cell-penetrating peptide amphiphile (CPPA) underwent self-assembly into nanofibers with long circulation times in blood. Upon accumulation in the tumor microenvironment, MMP-2 detached the sensitive linker, exposing the previously hidden CPP sequences on the surface of the assemblies and enhancing their cell-penetrating process. On the contrary, micelle-to-fiber morphological transitions studied by Ulijn and coworkers^89^ demonstrated their potential for sustainable chemotherapeutic drug release. A peptide amphiphile with MMP-9 responsiveness was synthesized and then self-assembled into spherical aggregates. DOX can be encapsulated into the hydrophobic inner core. After MMP-9 hydrolysis, the fiber forming moiety remained and re-assembled into nanofibers, providing a depot for sustainable drug release.

## 4 Immunotherapy

The clinical successes of checkpoint blockade antibodies have revolutionized immunotherapy for treatment of tumors. In contrast to traditional therapy, immunotherapy aims to boost the host immune system and then kill tumor cells in a safe and effective manner with minimal side effects. Peptide-based, tumor microenvironment-oriented self-assemblies have attracted tremendous attention in the respect of vaccine design and immunosuppressive microenvironment remedy.

### 4.1 Vaccine design

Modulating noncovalent interactions of peptide-based self-assemblies allows for formation of immune nanodrugs showing supramolecular self-adjuvant effects, which promoted antitumor immunotherapy. The pioneer work was reported by Collier and coworkers.^90^ Model antigen OVA_323–339_ was attached to a domain of Q11 (QQKFQFQFEQQ) to obtain peptide-based building blocks, OVA-Q11. Then, the OVA-Q11 self-assembled into nanofibers. *In vivo* results demonstrated that nanofibers increased the population of IgG titers in serum, thereby enhancing the antitumor immunotherapy. Further studies suggested that the immune response relied on conjugation of OVA_323–339_ and Q11. Li and coworkers^91^ also used the Q11 domain to conjugate epitopes. MUC1-derived epitopes with varied glycosylated threonine residues were conjugated to Q11 for the design of building blocks. Epitope-Q11 conjugates self-assembled into fibrous self-adjuvant vaccines. These strategies provided a novel vaccine candidate with a simple and chemically defined formulation. Our group^92^ also reported an antitumor immune hydrogel through electrostatic coupling between poly-l-lysine (PLL) and a dipeptide derivative (Fmoc-FF). The formed helical fibril hydrogels without addition of antigens, immune regulatory factors, and adjuvants, showed a promising perspective for the development of immune responsive nanodrugs for antitumor therapy.

Distinct from the methods mentioned above, Yang and coworkers^93^ created supramolecular hydrogels by virtue of enzyme-assisted self-assembly, and further suggested their self-adjuvant function. In detail, the enantiomeric effect was investigated by the synthesis of peptide gelators Nap-GFFpY-OMe and Nap-G^D^F^D^F^D^pY-OMe. Both of them can co-assemble with OVA for the formation of hydrogels upon ALP catalysis. Effective cellular cytotoxic immune responses of the two enantiomeric hydrogel adjuvants were verified. Particularly, d-peptide based hydrogels possessed superior accumulation in lymph nodes, thereby preventing tumor growth effectively. The work provided a facile strategy to administer vaccine adjuvants with biosafety.

### 4.2 Immunosuppressive microenvironment remedy

Coupling supramolecular self-assembly and the acidic tumor microenvironment may promote the release of immunoactive molecules, remedying the immunosuppressive microenvironment. For example, Xie and coworkers^94^ synthesized responsive exosome (Exo) bioconjugates for immunotherapy. Firstly, the macrophages were polarized to anti-tumoral 1 macrophages (M1) and the cell membranes were modified with azide groups. Then, the dibenzocyclooctynes (DBCO)-modified anti-CD47 antibody (aCD47) and anti-signal regulatory protein alpha antibody (aSIRPa) were conjugated to azide-modified M1 Exo linked by pH-sensitive benzoic-imine bonds. aCD47 oriented the M1 Exo to target tumor by bonding to CD47 on tumor cell membranes while the acidic microenvironment cleaved the benzoic-imine bonds to further release aSIRPa and aCD47. The inhibitory receptors of SIRPa and CD47 were individually blocked, thus improved the phagocytosis of macrophages. Simultaneously, M1 Exo re-educated the immune-suppressive M2 to immune-promotive M1 for tumor immunotherapy.

**Fig. 4:**
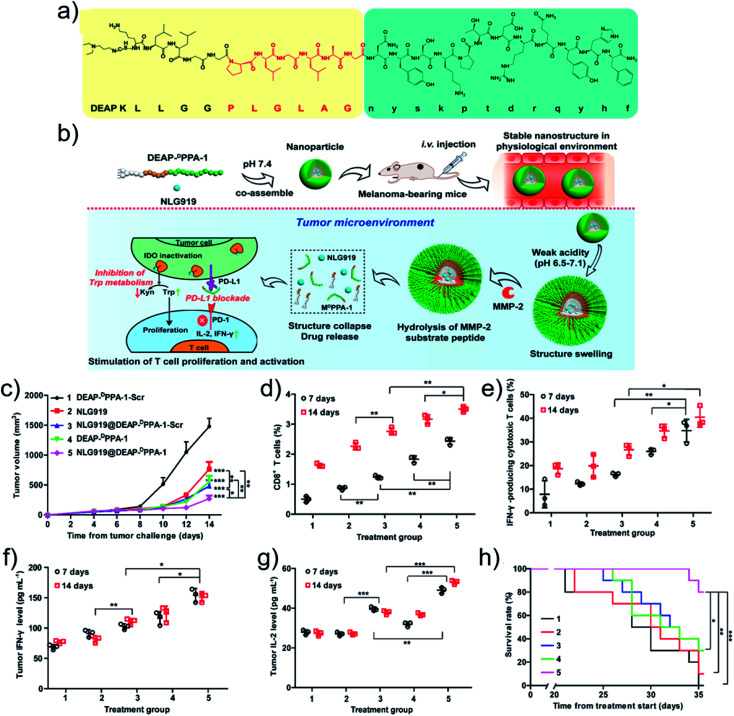
(a) Chemical structure and (b) antitumor mechanism of **DEAP-DPPA-1**. (c) Tumor volume profiles of mice. The ratio of (d) CD8^+^ T cells and (e) IFN-γ-producing CD8^+^ T cells. The level of (f) IFN-γ and (g) IL-2 cytokines. (h) Survival rate profiles of mice. Treatment groups: 1, **DEAP-DPPA-1**-Scr; 2, NLG919; 3, NLG919@**DEAP-DPPA-1**-Scr; 4, **DEAP-DPPA-1**; 5, NLG919@**DEAP-DPPA-1**. Reproduced with permission from ref. 52. Copyright 2018 American Chemical Society.

Other stimuli were also exploited to design smart nanodrugs for tumor immunotherapy. Liu and coworkers^95^ designed a GSH-responsive nanodrug. A positively charged cell-penetrating peptide with the sequence CWWR_8_CR_8_CR_8_C was synthesized and co-assembled with negatively charged OVA antigen upon electrostatic interactions. The oxidization of the thiols in the cysteine residues promoted the formation of disulfide bonds that cross-linked the structure to obtain stable peptide/OVA nanodrugs. Owing to the cell-penetrating ability of the peptide, the antigen uptake of the peptide/OVA nanodrugs was increased. After internalization, the antigen was released in the cytoplasm since the GSH cleaves the disulfide bonds, resulting in potent CD8^+^ T cell immunity. Alternatively, as an example based on enzyme-responsiveness, Nie and coworkers^52^ fabricated an immune nanodrug based on co-assembly of an amphiphilic peptide and NLG919, an inhibitor for immunosuppressive indoleamine 2,3-dioxygenase (IDO). The amphiphilic peptide contained a 3-diethylaminopropyl isothiocyanate (DEAP) segment, a PLGLAG domain with responsiveness to MMP-2, and a short D-peptide antagonist (^D^PPA-1). The resulting nanodrug (NLG919@**DEAP-DPPA-1**) sequentially responded to the tumor microenvironment (Fig. 4a). The acidic pH protonated the DEAP molecules and MMP-2 cleaved the PLGLAG segments, releasing ^D^PPA-1 and NLG919 for immunotherapy (Fig. 4b). For the testing of the antitumor efficacy, a similar amphiphilic peptide with a scrambled amino acid sequence (**DEAP-DPPA-1**-Scr) was synthesized as a negative control group. Compared to other groups, the NLG919@**DEAP-DPPA-1** nanodrug showed superior antitumor efficacy (Fig. 4c). As expected, the relative proportions of CD8^+^ T cells (Fig. 4d) and IFN-γ-producing cytotoxic T cells (Fig. 4e) were elevated. Consistently, higher productions of IFN-γ and IL-2 were obtained (Fig. 4f and g). In particular, the survival rates of mice were prolonged as well (Fig. 4h). These results demonstrated that the co-delivery of **DEAP-DPPA-1** and NLG919 can trigger robust antitumor immune response.

## 5 Combinational therapy

Monotherapy is normally insufficient to eradicate tumors because of metastasis and recurrence of tumor cells. Combinational therapy, that integrates multiple therapeutic agents into one platform for enhanced antitumor efficacy, is of particular interest.^96–98^ With peptide-modulated, tumor microenvironment-oriented self-assemblies for combinational tumor therapy, the synergistic therapeutic effect can be realized.

The promoted effect may be optimized by integrating therapeutic modalities.^99^ Hyperthermia caused by PTT can accelerate the blood circulation and increase oxygen perfusion, benefiting oxygen-dependent therapeutic modalities, such as PDT and radiotherapy.^14^ Intriguingly, phototherapies not only kill primary tumor cells, but also generate tumor debris for promoting an antitumor immune response, named “immunogenic cell death (ICD)”. ICD is associated with the release of damage-associated molecular patterns that are beneficial for antigen uptake and subsequent immune system activation.^100,101^ For instance, Qian and coworkers^102^ constructed multifunctional nanodrugs with the abilities of tumor-targeting, penetration enhancement, targeted drug release behavior, and immunomodulation mediated by checkpointed blockade (PD-1/PD-L1). Nanodrugs were co-assembled from a chemotherapeutic drug (docetaxel, DTX), photothermal agent (IR820) and a predesigned peptide with 27 amino acids (CF27). CF27 functioned as a cross-linker in the nanodrug fabrication to yield high drug loading efficiency. CF27 is responsive to MMP-2 and GSH in the tumor microenvironment, Significantly, CF27 contained a peptide sequence for blocking the PD-L1 immunosuppressive signal. The resulting nanodrug integrated photothermal therapy, chemotherapy and immunotherapy, strikingly inhibiting the tumor growth and metastasis.

Another typical example of combinational therapy was recently reported by Cui and coworkers.^103^ They utilized a drug-based supramolecular prodrug to achieve locally delivered immune checkpoint blockers (ICBs) for combination chemoimmunotherapy. By conjugating hydrophilic iRGD to two hydrophobic CPT molecules through an MMP-2 responsive peptide linker PLGLAG, the amphiphilic prodrug, diCPT-PLGLAGiRGD, was synthesized. In order to attach the CPT moiety and PLGLAG sequence, a reducible etcSS linker was applied to endow the amphiphilic prodrug with GSH responsiveness. The prodrug self-assembled into supramolecular nanotubes (P-NTs) in aqueous solution. Introducing PBS into the solution promoted the quick sol–gel transition for the formation of hydrogel due to the charge screening effect from the counterions. Simultaneously, by mixing the antibody of aPD1 with P-NTs, the P-NT-aPD1 hydrogel was obtained. *In vivo* results also demonstrated that localized delivery of aPD1 and a P-NT solution resulted in the formation of P-NT-aPD1 hydrogel within the injection site in mice. MMP-2 cleavage and GSH reduction in the tumor microenvironment promoted the degradation of P-NT-aPD1 hydrogel. Hence, the formed hydrogel played the role of a therapeutic reservoir for extended tumoral release of CPT and aPD1 which increased the frequency of *T*_effs_ and reduced the population of immune suppressor cells, thereby provoking a long-term immune response against tumor. Combined with the chemotherapeutic drug CPT, the tumor regression was regressed, and the tumor recurrence and metastasis were inhibited.

## 6 Conclusion

In summary, we have highlighted the recent advances of peptide-modulated, tumor microenvironment-oriented adaptive nanodrugs for improved tumor therapy. Programmable or functional peptides serving as building blocks regulate the self-assembly of drug molecules through multiple noncovalent interactions for fabrication of nanodrugs. Ubiquitous physiological variations in the tumor microenvironment, such as decreased pH, increased GSH and overexpressed enzymes, play the trigger roles to promote drug release or induce morphological transformation of nanodrugs and thereby improve the drug bioavailability and optimize the treatment outcome.

Notwithstanding the giant successes of peptide-modulated, tumor microenvironment-oriented adaptive nanodrugs that have been gained, the clinical translation remains a great challenge. Firstly, with peptides acting as regulating blocks, some chemical modifications, such as introducing amphiphilic moieties, are inevitable. Although structural stability has been realized, concerns about immunogenicity and biosafety have been raised. Secondly, the tumor entity is heterogeneous, which determines the complexity of interactions between nanodrugs and tumor cells. Understanding the tumor microenvironment by virtue of several physiological parameters is oversimplified. Thirdly, an ideal adaptive nanodrug requires considerable stability in blood circulation and ultra-sensitive responsiveness in the tumor microenvironment, which is still a formidable challenge for peptide-modulated self-assembly and other supramolecular self-assembly techniques. Regarding the challenges mentioned above, further improvements of nanodrugs are suggested as follows. First and foremost, to guarantee the functionality and biosafety, it is of significance to screen peptides with intrinsic bioactivity, explicit metabolic mechanism and multiple noncovalent interaction-donating groups.^104^ Besides, real-time monitoring of dynamic interactions between nanodrugs and tumor cells is important and urgent, which calls for in-depth understanding of tumor microenvironments and self-assembly mechanisms. Novel agents, methods and techniques should be developed to indicate the noncovalent interactions *in vitro* and *in vivo*. Last but not least, the stability and the responsiveness of nanodrugs should be optimized. Noncovalent interactions can be enhanced and manipulated by modulating thermodynamic or kinetic parameters. For example, applying metal coordination to stabilize the structural integrity in blood circulation is practical.^105^ Integrating complementary triggers into one platform improves the responsive sensitivity of nanodrugs in the tumor microenvironment. Combining and exploiting intracellular dysfunctional signals, such as ROS,^106^ may improve the responsive sensitivity on the site of interest and enhance the specificity of tumor inhibition. Overall, the collaboration of researchers with multidisciplinary backgrounds is needed to construct smart nanodrugs and accelerate their clinical applications.

## Conflicts of interest

There are no conflicts to declare.
